# Ni-Co bimetal nanowires filled multiwalled carbon nanotubes for the highly sensitive and selective non-enzymatic glucose sensor applications

**DOI:** 10.1038/srep36583

**Published:** 2016-11-11

**Authors:** K. Ramachandran, T. Raj kumar, K. Justice Babu, G. Gnana kumar

**Affiliations:** 1 Department of Physical Chemistry, School of Chemistry, Madurai Kamaraj University, Madurai-625021, Tamil Nadu, India

## Abstract

The facile, time and cost efficient and environmental benign approach has been developed for the preparation of Nickel (Ni)-Cobalt (Co) alloy nanowires filled multiwalled carbon nanotubes (MWCNTs) with the aid of mesoporous silica nanoparticles (MSN)/Ni-Co catalyst. The controlled incorporation of Ni-Co nanostructures in the three dimensional (3D) pore structures of MSN yielded the catalytically active system for the MWCNT growth. The inner surface of MWCNTs was quasi-continuously filled with face-centered cubic (fcc) structured Ni-Co nanowires. The as-prepared nanostructures were exploited as non-enzymatic electrochemical sensor probes for the reliable detection of glucose. The electrochemical measurements illustrated that the fabricated sensor exhibited an excellent electrochemical performance toward glucose oxidation with a high sensitivity of 0.695 mA mM^−1^ cm^−2^, low detection limit of 1.2 μM, a wide linear range from 5 μM–10 mM and good selectivity. The unprecedented electrochemical performances obtained for the prepared nanocomposite are purely attributed to the synergistic effects of Ni-Co nanowires and MWCNTs. The constructed facile, selective and sensitive glucose sensor has also endowed its reliability in analyzing the human serum samples, which wide opened the new findings for exploring the novel nanostructures based glucose sensor devices with affordable cost and good stability.

The design and development of novel glucose sensors with high sensitivity, reliability, fast response, good selectivity and low-cost have received tremendous importance in variety of fields including clinical diagnostics, ecological and food analysis, bioprocess control, pharmaceutical analysis etc.,^1^,^2^,^3^. Diabetes has become a global endemic and its patient population will drastically increase in the forthcoming years^4^. The complications of diabetes including coronary artery, peripheral vascular disease, stroke, renal failure and blindness could be controlled by the tight monitoring of blood glucose levels, in which the role of electrochemical technique is pivotal^5^. The instrumental simplicity, moderate cost and portability of electrochemical sensors increased the occupancy of entire biosensors market to 85%^6^. Initially, the primary development of electrochemical quantification of glucose was vastly relied upon the performances of specific biocatalysis of enzymes including glucose oxidase (GOx) and glucose dehydrogenase (GDH). The definite immobilization of enzymes and direct exposure of their redox active sites toward glucose offered the sufficient sensitivity and selectivity, which were optimal for the clinical practice in diabetes management^7^,^8^. However, the denaturation of enzymes during the electrode fabrication, storage and exploitation, high sensitivity toward temperature, pH and humidity, poor reproducibility and high cost, turn out to be the significant constrains of enzymatic glucose sensors, which directed the research domains toward the exploration of non-enzymatic glucose sensors comprising metal nanocatalysts. However, the agglomeration, weaker electrical conductivity, poisoning and fouling toward the chemisorbed intermediates and mechanical instability of metal nanocatalysts significantly impeded the wide spread applications of non-enzymatic glucose sensors, which could be substantially tackled with the composite formation of nanocatalysts with the active carbon materials^9^.

Carbon based nanomaterials have been widely exploited for the fabrication of electrochemical sensors on account of their low cost, electrical conductivity and excellent corrosion resistance in various electrolytes^10^. In specific, multiwalled carbon nanotubes (MWCNTs) with the helical tubular structures exhibit large surface area, high sensitivity, rapid electron transfer rate etc., which triggered their extensive utilization in electrochemical sensors, demanding its production with high purity^11^,^12^. In general, MWCNTs are produced by the chemical vapordeposition (CVD) technique during the pyrolysis of hydrocarbon gases at high temperatures and is considered as one of the most efficient ways to obtain high purity CNTs with unique properties^13^. The yield, growth dimensions and other unique properties of MWCNTs could be effectively tailored by the precursor gas, flow rate, temperature and catalyst. In general, the preparation of MWCNTs is achieved with the aid of catalysts and the carbon precursor molecules are catalytically decomposed on the surface of catalysts, resulting the incorporation of carbon atoms into the catalyst^14^. Once the supersaturation is occurred, carbon atoms will precipitate from the catalyst, leading to the growth of nanotubes. It is widely accepted that the size and chemical composition of metal nanoparticles effectually determine the diameter and structural perfection of the nanotubes^15^,^16^. Hence, number of efforts has been devoted on the transition metal catalysts such as Fe^17^, Co^18^, Ni^19^, Mo^20^ and Cr^21^ and its bimetals^22^ for the preparation of MWCNTs. Recently, the bimetallic nanostructures with two different cations have received keen interest as catalysts in the preparation of controlled diameter and enhanced yield of MWCNTs^23^. It is understood that two stages of mechanism are involved in the preparation of MWCNTs; one metal is responsible for the nucleation process, while the other is responsible for the catalytic growth^24^.

However, the bimetal nanostructures may aggregate under high temperatures, which is highly inactive for the uniform growth of MWCNTs. The diameter of catalyst matrix is closely correlated with the diameter of MWCNTs, leading to the interest on well dispersed nanosized catalyst systems with the narrower size distribution^25^. Hence, a keen focus has been paid toward the active substrate supported catalysts and the metal-substrate interactions play a significant role in determining the growth of MWCNTs, which directed the exploitation of various catalyst supports including Zeolite^26^, Al_2_O_3_^27^, SiO_2_^28^, TiO_2_^29^, CaCO_3_^30^, mesoporous silica nanoparticles (MSN) etc., Among the aforesaid active supports, MSN is considered as the ideal choice for the synthesis of MWCNTs, owing to its uniform and tailorablemesopores, large surface area, narrow pore size distribution, ordered pore structure, biocompatibility etc.,^31^. The inactive siliceous properties construct the ideal interaction with the active metal nanoparticles along with the less interruption by the metal-support interaction^32^. The growth and orientation of MWCNTs could be perfectly tuned and templated by the regularly ordered MSN substrate.

Although MWCNTs exhibited the high surface to volume ratio, it is not capable of electrooxidizing glucose, owing to the absence of catalytic active sites^33^, requiring the incorporation of metal nanocatalysts. In general, the metal nanoparticles are anchored in outer shells of commercially available MWCNTs and exploited as the electrochemical probes in non-enzymatic detection of glucose. Pt nanocatalysts anchored over the acid-functionalized MWCNTs exhibited the sensitivity of 20.1 mAM^−1^cm^−2^ with a linear range of 1.2 μM to 8.4 mM toward the electrochemical oxidation of glucose^34^. Ni nanoparticles deposited on acid-treated MWCNTs demonstrated a linearity range of 3.2 μM to 17.5 mM, detection limit of 0.89 μM and sensitivity of 67.2 μAmM^−1^ cm^−2^ toward the electrochemical detection of glucose^35^. MWCNT/Co composite prepared by using an electrodeposition technique under the acidic medium exhibited a lower detection limit of 0.009 μM with the sensitivity of 727 μAmM^−1^cm^−2^ and linear range of 0.005 to 1 mM^36^.

From the reported literatures, it is clear that the deposition of metal nanoparticles on the outer shells of MWCNTs necessitated the acid-functionalization, which may hamper their applications in biosensors. These conventional strategies cause the structural damage to the walls of MWCNTs, leading to the loss of their electrical conductivity and mechanical properties^37^. The loss of mechanical properties leads to the uneven distribution of metal nanoparticles over the walls of MWCNTs, resulting the non-interconnected conduction channels for the glucose detection and the involved preparation procedures are complicated multi-step processes^38^. Hence, the exploitation of inner cavity of MWCNTs as a nanoreactor is an exciting possibility to tackle the aforementioned significant issues and the metal nanoparticles filled MWCNTs could enhance their catalytic activity along with the high resistance toward sintering process^39^. When CNTs are filled with the metal nanoparticles, an unique host-guest electronic interaction will be exerted, which may change the local work function of MWCNTs walls^40^. However, a significant research effort has not been devoted to explore the influence of metal nanoparticles filled MWCNTs toward the electrochemical applications. Hence, this report is aimed to develop an efficient strategy to prepare the Ni and Ni-Co bimetal nanowires filled MWCNTs without damaging the shape, structure and walls of MWCNTs. It is also envisaged to identify the influential parameters involved in the effectual electrooxidation of glucose under various electrochemical regimes and conditions.

## Results

### Morphological properties

**Figure 1:**
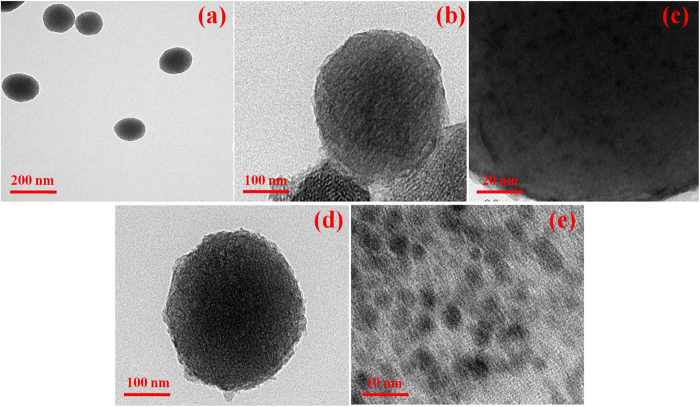
TEM images of (**a**) MSN, (**b**,**c**) MSN/Ni and (**d**,**e**) MSN/Ni–Co nanostructures.

The morphological images of as-prepared MSNs exhibited the highly ordered mesoporous network structure with the hexagonal arrays (Fig. 1a). The pore walls enabled pore connectivity with the large surface area and narrow pore size distribution and the average particle size of MSNs is found to be 150 nm. From Fig. 1b–e, it is clear that Ni and Ni-Co nanoparticles, respectively, were effectively confined in the mesoporous channels of MSN.

**Figure 2:**
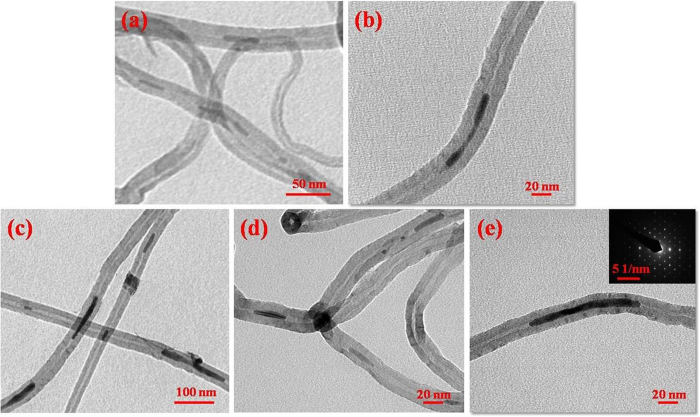
TEM images of (**a**,**b**) MWCNT/Ni and (**c**–**e**) MWCNT/Ni-Co nanostructures (inset **e**: SAED pattern of Ni-Co nanowire).

The transmission electron microscopic images of as-prepared MWCNT/Ni and MWCNT/Ni-Co nanostructures are depicted in Fig. 2. Although the fine porous structure of MSN played an important role in the formation of MWCNTs, the pore structures of bare MSN were not influential enough for the growth of MWCNTs, compelling the utilization of active metal sites. Hence, the Ni/Ni-Co metal nanostructures anchored MSNs were subjected for the CVD treatment for the effectual growth of MWCNTs. The as-prepared nanostructures exhibited the uniform shape of tubular like morphologies with the thick tube walls of MWCNTs. The average diameter and length of MWCNTs were found to be 38 nm and several micrometers, respectively, and the average tubular inner diameter of MWCNTs was measured to be 13 nm (Fig. 2). The most of MWCNTs in MWCNT/Ni composite were filled with Ni nanowires (Fig. 2a,b) and the length and average diameter of filled Ni metal nanowires are found to be several hundreds of nanometers and 13 nm, respectively (Fig. 2a,b). The filled Ni nanowires were tightly wrapped by the nanotubular walls and were located in the centre of tubes. The morphological features and diameter observed for the Ni-Co nanowires filled MWCNTs (Fig. 2c–e) are identical with the Ni nanowires filled MWCNTs (Fig. 2a,b). The selected area electron diffraction (SAED) pattern of MWCNT/Ni-Co specified the face-centered cubic (fcc) structure of Ni-Co bimetal nanowires (Inset: Fig. 2e). The energy dispersive X-ray spectroscopy (EDAX) analysis of MWCNT/Ni-Co confirmed the presence of Ni-Co bimetal nanostructures in the prepared composite (Fig. S1).

### Structural characterizations

**Figure 3:**
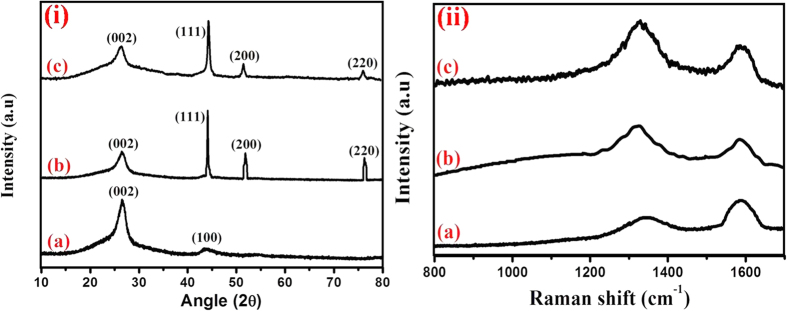
(i) XRD patterns and (ii) Raman Spectra of (**a**) MWCNT (**b**) MWCNT/Ni and (**c**) MWCNT/Ni-Co nanostructures.

MWCNTs exhibited a strong diffraction peak at 2θ = 26.5°, and a weak peak at 2θ = 43.79° (Fig. 3i-a), specifying the (002) and (100) reflection planes, respectively, of typical graphite^41^. In addition to the carbonaceous peaks, MWCNT/Ni exhibited the significant diffraction peaks at 2θ = 44.5, 51.9 and 76.3° (Fig. 3i-b), which can be indexed to the (111), (200) and (220) crystal planes of fcc structure of Ni, ensuring the presence of metallic Ni in MWCNT/Ni^31^,^42^. The observed reflection planes of Ni-Co bimetal in MWCNT/Ni-Co (Fig. 3i-c) are similar to the characteristic planes of Ni nanoparticles in the MWCNT/Ni, owing to the close lattice parameters of both Ni(3.523 °A) and Co(3.544 °A) (JCDPS 15-0806,Co;04-0850,Ni)^31^,^43^. The mean crystallite sizes of Ni and Ni-Co nanowires filled in the MWCNTs are estimated to be 13 nm, which are in good agreement with the obtained TEM images.

All of the MWCNTs based nanostructures exhibited the characteristic carbonaceous D and G Raman bands at 1336 and 1588 cm^−1^, respectively(Fig. 3ii). The D band is ascribed to the breathing modes of k-point photons of A_1g_ symmetry, whereas G band is attributed to the E_2g_ stretching mode of graphite^44^. The Ni nanowire filled MWCNTs (Fig. 3ii-b) exhibited the I_D_/I_G_ ratio of 1.32, which is higher than that of commercial MWCNTs (0.74) (Fig. 3ii-a). The I_D_/I_G_ ratio was further increased to 1.50 for the MWCNT/Ni-Co nanocomposite (Fig. 3ii-c), owing to the bimetallic effect of Ni and Co, representing the enhanced number of structural defects, increased localized sp^3^ defects in sp^2^ framework, high electrical conductivity, and decreased in-plane sp^2^ domains of MWCNT/Ni-Co composite.

The electrical conductivity of commercial MWCNTs is found to be 5.2 Scm^−1^. The nanometric cylindrical graphene sheets in the form of MWCNTs and its good electrical contact with the Ni nanowires enhanced the electrical conductivity to 16.3 Scm^−1^ for the MWCNT/Ni composite and was further increased for MWCNT/Ni-Co composite to 22.5 Scm^−1^.

### Electrochemical studies

**Figure 4:**
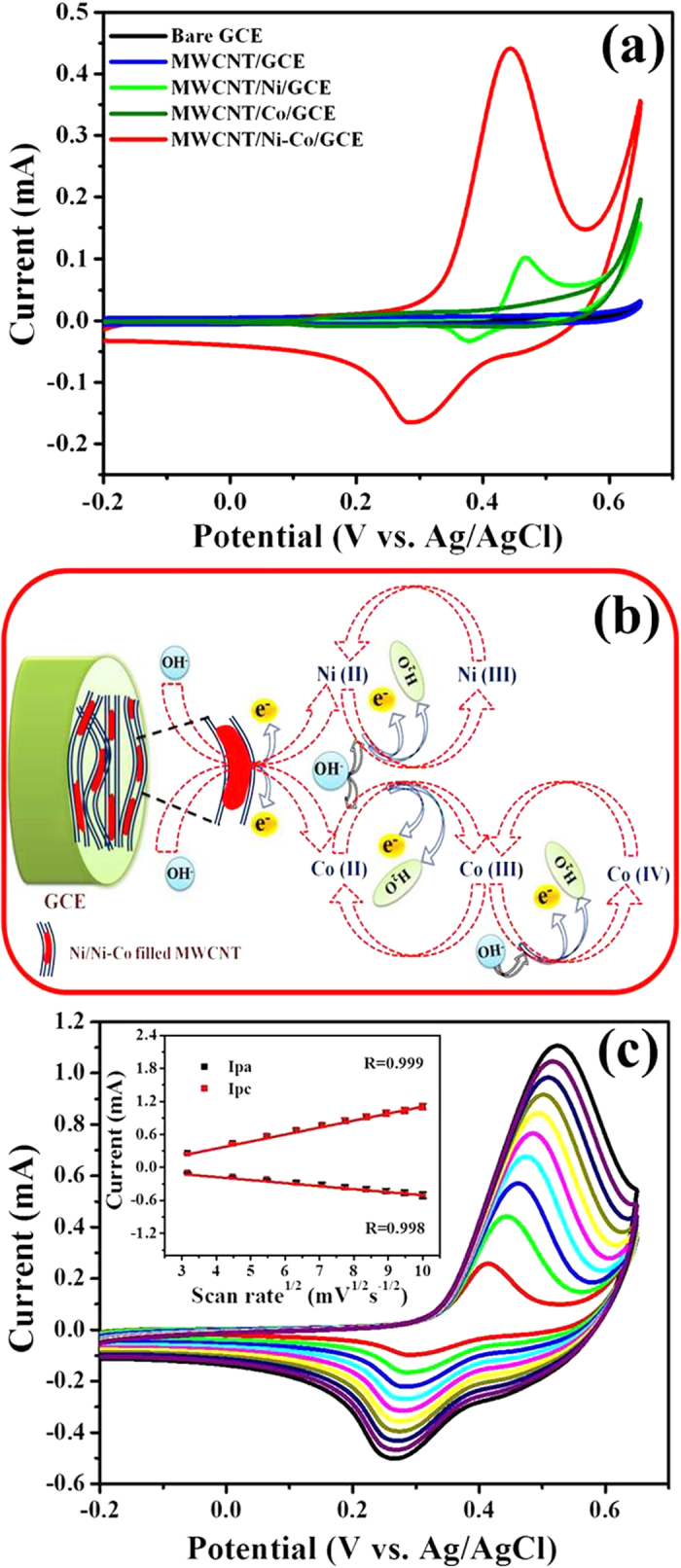
(**a**) Cyclic voltammograms of studied GCEs in 0.1 M NaOH at a scan rate of 20 mVs^−1^, (**b**) mechanism involved in the activation of MWCNT/Ni-Co using NaOH solution and (**c**) cyclic voltammograms of MWCNT/Ni–Co/GCE in 0.1 M NaOH with different scan rates ranging from 10 to 100 mV s^−1^ (inset: plot of square root of scan rate *vs*. peak current).

The electrochemical behaviors of studied Glassy Carbon Electrodes (GCEs) were investigated in 0.1 M NaOH (pH = 13) solution by using CV technique at a scan rate of 20 mVs^−1^ (Fig. 4a). The bare GCE has not exhibited any characteristic redox peaks, specifying the passive behavior of GCE. Although the increased background current was observed at MWCNT/GCE due to the elevated electrical conductivity and surface area, significant redox currents were not observed, owing to the absence of metallic active sites. In contrast, MWCNT/Ni/GCE exhibited the well-defined quasi reversible redox peaks, specifying the variation in the oxidation state of Ni nanostructures. Under the alkaline conditions, Ni(0) was combined with OH^−^ ions and generated Ni(II). It was further proceeded by the oxidation of Ni(II)/Ni(III) in the anodic sweep (Epa) at 0.46 V *vs*. Ag/AgCl and the cathodic sweep (Epc) found at 0.37 V *vs*. Ag/AgCl is ascribed to the Ni(III)/Ni(II) reduction^45^. MWCNT/Co/GCE displayed two Epas at 0.18 and 0.52 V *vs*. Ag/AgCl, corresponding to the oxidation of Co(II)/Co(III) and Co(III)/Co(IV), respectively. The Epcs of MWCNT/Co/GCE located at 0.13 and 0.44 V *vs*. Ag/AgCl are associated with the reduction of Co(IV)/Co(III) and Co(III)/Co(II), respectively^46^.

The improved redox behavior was observed at MWCNT/Ni-Co/GCE as evidenced from the higher peak currents in comparison with MWCNT/Ni/GCE, which might be attributed to the improved electrical conductivity and large surface area of MWCNT/Ni-Co composite, which are provided *via* the synergetic effects of Ni and Co metal nanostructures. The well-defined Epa observed at 0.45 V *vs*. Ag/AgCl is attributed to the transformation of different complex oxidation species of Ni-Co in the alkaline medium. The Ni(II) and Co(II) formed under the alkaline conditions were transformed into Ni(III) and Co(III), respectively, in which Co(III) was further oxidized into Co(IV) at higher potentials. Under the reverse scan, Ni(III) and Co(IV) were electrochemically reduced into Ni(II) and Co(III), respectively^47^, and the involved electrochemical reactions are schematically illustrated in Fig. 4b.

Figure 4c depicts the CV responses of MWCNT/Ni-Co/GCE in 0.1 M NaOH solution (pH = 13) as a function of scan rate ranging from 10–100 mVs^−1^. It can be seen that both the Ipa and Ipc values were gradually increased with an increase in the scan rate, whereas the positive and negative shifts were noticed for Epa and Epc, respectively. Furthermore, the peak currents of both oxidation and reduction reactions are linearly scaled with the square root of scan rate with the high correlation coefficients(Inset: Fig. 4c).

**Figure 5:**
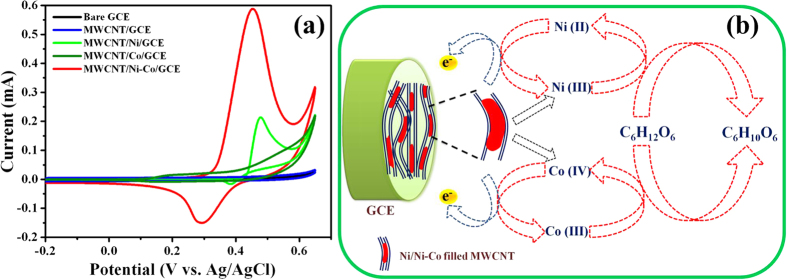
(**a**) Cyclic voltammograms of studied GCEs in 0.1 M NaOH in the presence of 2 mM glucose at a scan rate of 20 mV s^−1^ and (**b**) mechanism involved in the electrooxidation of glucose at MWCNT/Ni-Co.

The electrocatalytic activities of fabricated GCE, MWCNT/GCE, MWCNT/Ni/GCE, MWCNT/Co/GCE and MWCNT/Ni-Co/GCE were investigated by using CV technique in 0.1 M NaOH (pH = 13) solution in the presence of 2 mM glucose at a scan rate of 20 mV s^−1^ (Fig. 5a). Even under the presence of an analyte, bare GCE and MWCNT/GCE demonstrated only the background currents, specifying their passive behavior toward glucose oxidation. However, MWCNT/Ni/GCE exhibited the prompt oxidation current (Ipa) of 0.21 mA at 0.47 V *vs*. Ag/AgCl, specifying the involvement of Ni(II)/Ni(III) redox couple toward the glucose electrooxidation. The analyte glucose (C_6_H_12_O_6_) was oxidized into gluconolactone (C_6_H_10_O_6_) through Ni(III) in an alkaline medium, as evidenced from the increased Ipa values^31^,^45^,^48^. The electrooxidation of glucose at MWCNT/Co/GCE was achieved *via* the Co(III)/Co(IV) redox couple at 0.53 V *vs*. Ag/AgCl^46^ and the observed electrooxidation process is inferior to MWCNT/Ni/GCE as substantiated from the obtained lower Ipa of 0.09 mA. On the other side, MWCNT/Ni-Co/GCE demonstrated an increased Ipa of 0.58 mA at 0.43 V *vs*. Ag/AgCl, which is much higher than that of MWCNT/Ni/GCE and MWCNT/Co/GCE. The Ni(III) and Co(IV) generated under an alkaline medium effectively oxidized glucose into glucanolactonane (Fig. 5b) and the improved glucose electrooxidation process than that of monometallic Ni or Co counterpart is purely attributed to the bimetallic effect^31^,^47^,^48^.

**Figure 6:**
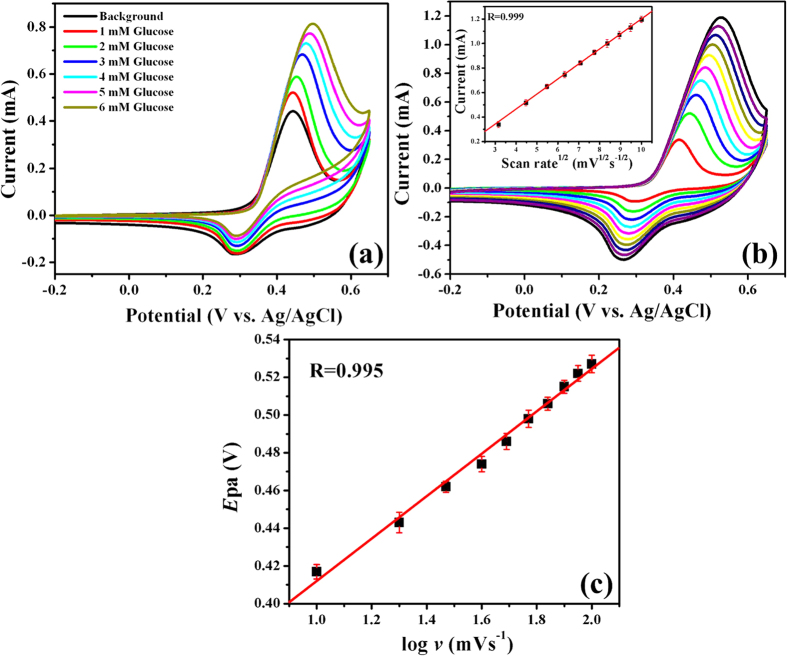
(**a**) Cyclic voltammograms of MWCNT/Ni–Co/GCE in 0.1 M NaOH with different concentrations of glucose at a scan rate of 20 mV s^−1^, (**b**) cyclic voltammograms of MWCNT/Ni–Co/GCE in 0.1 M NaOH in the presence of 2 mM glucose with different scan rates ranging from 10 to 100 mV s^−1^ (inset: plot of square root of scan rate *vs*. peak current) and (**c**) Epa *vs*. logarithm of scan rate for the oxidation of 2 mM glucose at MWCNT/Ni–Co/GCE.

Figure 6a shows the voltammograms of MWCNT/Ni-Co/GCE in 0.1 M NaOH solution as a function of glucose concentration ranging from 1–6 mM at a scan rate of 20 mV s^−1^. Upon the increment in glucose concentration, the gradual enhancement in Ipa associated with the positive shifts in Epa were observed. Furthermore, Ipcs were decreased with an increase in the glucose concentrations, owing to the consumption of Ni(III)/Co(IV) in the electrooxidation of glucose. The prominent glucose electrooxidation behaviour observed at MWCNT/Ni-Co/GCE as a function of glucose without any fouling effect offered an excellent platform for the amperometric i-t determination of fabricated sensors^47^,^48^.

Figure 6b reveals the voltammograms of MWCNT/Ni-Co/GCE containing 2 mM glucose in 0.1 M NaOH solution, specifying that the electrooxidation of glucose at MWCNT/Ni-Co/GCE is a quasi-reversible electrochemical process. It is evident that Ipa was linearly enhanced with the square root of scan rate (*v*^1/2^) with a high correlation coefficient of 0.999, which provided a linear Ipa-*v*^1/2^ relationship. Since the quasi-reversible electrochemical process is intrinsically affected by the electrode reaction kinetics at a CV peak potential, it is difficult to claim that the quasi-reversible electrode process is a diffusion-controlled on the basis of Ipa-*v*^1/2^ relationship and it is found that the Epa was positively shifted with an increase in the scan rate (Fig. 6b). The peak potential is significantly concerned in CV experiments, however, the potential is stepped to a very high anodic potential in a potential-step experiment. Hence, it is concluded that the quasi-reversible anodic reaction achieved at MWCNT/Ni-Co/GCE may be a diffusion controlled process, owing to the provision of a high electrical energy into the electrolytic system to effectively overcome the activation energy of the quasi-reversible anodic reaction^49^. To further confirm the diffusion controlled process of glucose oxidation at MWCNT/Ni-Co/GCE, the relationship of log Ipa *vs*. log *v* was established (Fig. S2). The log Ipa-log *v* showed a linear relationship with the high correlation coefficient of 0.999 and the slope of logarithmic curve is found to be closer to 0.5, specifying that the reaction is a diffusion controlled process^50^.

An increase in the scan rate has not only increased the Ipa but has also positively shifted Epa values toward the electrooxidation of glucose, specifying that a kinetic limitation was involved in the reaction between redox sites of MWCNT/Ni-Co and glucose (Fig. 6c). The charge transfer coefficient (α) for the involved reaction is evaluated by using the following equation of Epa = 0.0113log*v* + 0.298 with the correlation coefficient of 0.995. According to Laviron theory^51^, the slope of the plot of Epa *vs*. logarithm of scan rate is 2.3RT/(1 − α)nF and α is found to be 0.52.

**Figure 7:**
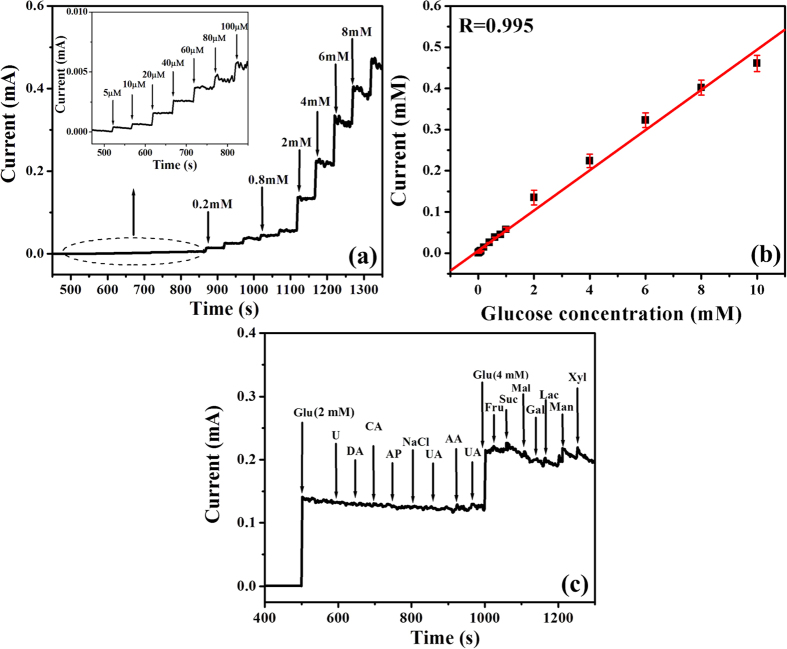
(**a**) Amperometric responses of MWCNT/Ni–Co/GCE upon the successive injections of glucose in 0.1 M NaOH at an applied potential of 0.45 V *vs*. Ag/AgCl (inset: amperometric responses of MWCNT/Ni–Co/GCE toward 5 to 100 μM concentration of glucose), (**b**) calibration plot of MWCNT/Ni–Co/GCE amperometric current responses as a function of glucose concentration and (**c**) interference test of MWCNT/Ni-Co/GCE with the successive addition of glucose and other interfering species in 0.1 M NaOH at an applied potential of 0.45 V *vs*. Ag/AgCl.

The accurate detection of glucose atMWCNT/Ni-Co/GCE was carried out by using amperometric technique with the successive injection of a known concentration of glucose into a stirring 0.1 M NaOH solution at an applied potential of 0.45 V *vs*. Ag/AgCl (Fig. 7a). It was observed that after an each addition of glucose solution, a notable enhancement of current response was rapidly obtained and the steady-state current was achieved within 5 s. MWCNT/Ni-Co/GCE responded linearly to the glucose concentration ranging from 0.005 to 10 mM with a high correlation efficient of 0.995. From the calibration curve (Fig. 7b), the sensitivity and detection limit of MWCNT/Ni-Co/GCE toward glucose oxidation were calculated to be 0.695 mAmM^−1^cm^−2^ and 1.2 μM, respectively, and the detection limit was estimated by using the formula 3 sb/S, where sb = standard deviation of the background current and S = slope of calibration plot^52^. The high signal noise observed for MWCNT/Ni-Co/GCE at higher glucose concentration is attributed to the accumulation of adsorbed intermediate species on the electrode surface. However, the response current and time observed at higher glucose concentration are in good agreement with the lower glucose concentration, which denoted the robust stability of MWCNT/Ni-Co/GCE under the harsh electrochemical regimes and conditions.

## Discussions

The Ni and Ni-Co bimetal nanoparticles decorated MSN were exploited as the catalytic templates for the effectual growth of MWCNT/Ni and MWCNT/Ni-Co composites, respectively. The Ni^2+^ and Co^2+^ ions generated from the respective precursor materials were electrostatically interacted with the negatively charged MSN and produced the intermediate Si-O-Ni^2+^ and Si-O-Ni^2+^-Co^2+^ bonds^31^,^53^. The subsequent reduction achieved *via* hydrazine lead to the formation of Si-O-Ni^0^ and Si-O-Ni^0^-Co^0^ composite and Ni/Ni-Co nanoparticles exhibited a narrow size distribution with an average diameter of 5 nm.

The growth of Ni/Ni-Co nanowire filled MWCNTs is found to be the catalytically controlled process. Initially, the metal nanoparticles embedded in MSN were fixed to the substrate and the tubes were grown toward the upward direction with an open-ended type, favoring the root growth mode. Under the high temperature, hydrocarbons were degenerated into carbon and hydrogen gases. Owing to the small size of Ni/Ni-Co nanoparticles, the melting point of Ni/Ni-Co is lower than that of their bulk counterpart, confirming that the catalysts would like to behave as a liquid in the growth stage. Under the liquid state, Ni/Ni-Co nanoparticles were agglomerated into a larger size and the formation of hydrogen may provide additional supports to the activation and segregation of a catalyst on the substrate. The diffusion of carbon atoms into the metal liquid clusters formed a cylinder over them and the nanotube growth was started after the super saturation of carbon deposition on the metal nanoparticles(Fig. S3)^54^. Under the high temperature, Ni/Ni-Co nanoparticles placed inside the nanotubes coalesced together and generated nanowires and CNTs and Ni/Ni-Co nanowires were consecutively grown under the carbon segregation. It is also evident that the filling of metal nanowires was decreased from the root to the top end of the MWCNTs, owing to the higher growth rate of carbon shells in comparison with the metal nanowires. The metal nanostructures were encapsulated at the ends of MWCNTs and the diffusion of metal nanoparticles in to the inner cavity of MWCNTs to reach the other nanoparticles has become tedious after a certain period. Once the distance between filled metal nanowires and MWCNTs was decreased, the growth rate of MWCNTs was also decreased, leading to the closure of free standing ends.

Although a similar mechanism was adopted for both of the MWCNT/Ni, MWCNT/Co and MWCNT/Ni-Co nanocomposites, the yield of MWCNT/Ni-Co (293%) assisted by the MSN/Ni-Co catalyst is higher than that of MWCNT/Ni (185%) and MWCNT/Co(160%) aided by the MSN/Ni and MSN/Co catalysts, respectively. The Ni-Co bimetal nanoparticles exhibited the greater ability toward the decomposition of acetylene molecules *via* the synergetic effects of two metals. The bimetallic compositional effect on catalytic performance may be related to the carbon mobility and diffusion, which effectively facilitated the CNT nucleation and growth^24^,^55^.

The tubular structure of MWCNTs provided larger surface area that facilitated the accommodation and diffusion of glucose toward the active sites without any potential barrier and facilitated the intimate contact between the electrode surface and analyte. The electrocatalysis process is usually achieved with the adsorption of an analyte with the electrode surface. The vacant ‘d’ orbitals and unpaired ‘d’ electrons associated with Ni(III) provided an unique electrocatalytic effect to the MWCNT/Ni composite, which generated the bond formation with the adsorbed analyte^31^. The higher adsorption and followed by the diffusion of an analyte provided the maximum availability to the metallic centres and the effectual electrooxidation of glucose was facilitated at the said active centres of MWCNT/Ni.

Owing to the open ended tubular morphology of MWCNTs, large surface area and increased number of active sites in MWCNT/Ni-Co composite, the electrode-electrolyte contact area of MWCNT/Ni-Co/GCE was promoted *vi*a the improved adsorption and diffusion of an analyte. Furthermore, MWCNTs exhibited the number of nanometer sized pores on its surfaces, which were advantageous for the inward diffusion of an analyte *via* the capillary siphon action into the hollow channels/inner surface of MWCNTs that maximized the direct contact between the analyte and metallic active sites and the involved mechanism is schematically illustrated in Fig. S4^56^. The tight packing of Ni nanowires in the inner surface of MWCNTs and its strong interaction with the graphitic shells facilitated the continuous and rapid electron transfer from metal to the graphitic walls. The theoretical studies on CNTs specified that the interaction of metal clusters with the internal surface of MWCNTs provided the stronger positive electrostatic environment and transferred number of electrons to the graphitic walls with the high electron density on MWCNTs surfaces in comparison with the exterior interaction of metal clusters with MWCNTs^57^. The encased metal nanoparticles activated the surrounding graphitic layers, leading to the alteration in the electronic structure and reduced the surface work function of graphitic layers, consequently the electrocatalytic activity of graphitic walls was enhanced. MWCNTs architecture has not only provided the good conducting matrix to maintain the structural integrity but has also enabled the reduction of interfacial resistance, which increased the rapid electron transport for the efficient electrooxidation process^58^. The Ni-Co nanowires filled MWCNTs effectively hindered the agglomeration and leach out of Ni nanowires from the MWCNTs, which greatly preserved the intrinsic electrical conductivity of the composite. The mechanism involved in the electrooxidation of glucose at MWCNT/Ni-Co nanostructures is schematically illustrated in Fig. 5b. The obtained analytical performances of fabricated MWCNT/Ni-Co/GCE sensor are comparable and even superior with the previously reported literatures (Table S1), guaranteeing its prospective applications.

The relative standard deviation (RSD) of 3.4% was observed for the eight identically fabricated MWCNT/Ni-Co/GCEs in 2 mM glucose in 0.1 M NaOH solution, guaranteeing the good reproducibility of fabricated sensors. MWCNT/Ni-Co/GCE retained 94.8% of initial amperometeric response even after 30 days toward the electrooxidation of glucose in alkaline solution. The excellent repeatability of fabricated sensor was observed from the obtained RSD of 3.8% for a set of seven measurements for a single MWCNT/Ni-Co/GCE, demonstrating its potential applications in the non-enzymatic glucose detection field.

The most significant analytical challenge of non-enzymatic glucose analysis is the discrimination of responses generated by the interference species, which have the similar electroactivity to the target analyte^59^. Although the physiological level of glucose (3–8 mM) is substantially higher than that of easily oxidizable interferences including ascorbic acid(AA) (0.1 mM) and uric acid(UA) (0.1 mM) in human blood, the electron transfer rates of aforesaid interfering species are higher than that of glucose and their oxidation responses are comparable to glucose^60^. Hence, the interference test of MWCNT/Ni-Co/GCE was evaluated with the successive addition of 2 mM glucose and 0.2 mM of interfering species such as urea (U), dopamine(DA), citric acid(CA), acetaminophen(AP), sodium chloride(NaCl), AA and UA in 0.1 M NaOH solution at an applied potential of 0.45 V *vs*. Ag/AgCl (Fig. 7c). MWCNT/Ni-Co/GCE exhibited an obvious response toward glucose oxidation, while insignificant responses were observed for the interfering species. Under the higher alkaline conditions (pH = 13), Ni-Co alloy exhibited an isoelectric point of 9.5–10.5, implying the negative charge of Ni-Co surfaces. Meanwhile, UA and AA exhibited the negative charges under the alkaline conditions, owing to the loss of protons, which exhibited the repelling effects against the negatively charged Ni-Co alloy nanostructures filled MWCNTs, providing an excellent selectivity toward glucose^31^. MWCNT/Ni-Co/GCE has also exhibited an excellent selectivity against the commonly found reducing sugars in human blood including 0.4 mM of fructose(Fru), sucrose(Suc), maltose(Mal), galactose(Gal), lactose(Lac), mannose(Man) and xylose(Xyl) and their concentrations are 10 fold lower than that of glucose(4 mM) and the maintained concentration ratio is in good agreement with the physiological level composition of glucose and other reducing sugars. Furthermore, the amperometric responses of MWCNT/Ni-Co/GCE toward glucose oxidation were not affected even under the presence of other interfering species, providing the excellent applicability of prepared electrochemical probes in accurate and highly selective glucose sensors.

The analytical reliability of MWCNT/Ni-Co/GCE was monitored with the human blood serum collected from a healthy volunteer and the concentration of glucose in the collected human blood serum is found to be 9.60 mM by using ACCU-CHEK Active glucometer. The collected blood serum sample was diluted with 0.1 M NaOH and pH level of the solution was maintained to be 13. The known concentration of glucose was gradually added into the blood serum and its amperometric responses were evaluated at an applied potential of 0.45 V *vs*. Ag/AgCl. The practical applicability of fabricated MWCNT/Ni-Co/GCE was validated with the recovery of 98.33–100.61% and 1.97**–**2.93% relative standard deviation (Table 1), which is in good agreement with the commercially available glucometer, specifying that the fabricated sensor could be used for the routine anlaysis of glucose in real biological samples.

In summary, Ni-Co bimetal nanoparticles were self assembled into ultra-long and thin nanowires within the confined space of MWCNTs *via* a one-step CVD technique on the decomposition of hydrocarbons on MSN/Ni-Co substrate and the prepared composite exhibited an admirable bio-sensing performances toward glucose without the backing of enzyme. The quasi-continuously filled Ni-Co nanowires in the inner surface of MWCNTs and graphitic structure of MWCNTs paved continuous path ways for the rapid electron transference. The intimate contact exerted between the analyte and Ni-Co metallic active sites achieved *via* the pore channels of MWCNTs lowered the interfacial charge transfer resistance and substantiated their higher electrocatalytic response toward glucose oxidation with the number of active features such as high sensitivity, long-term stability, selectivity, lower detection limit and good reproducibility. The outer surface of graphitic walls of MWCNTs protected the Ni-Co metal nanowires from agglomeration and increased their resistivity toward the surface poisoning of chemisorbed intermediates and guaranteed its analytical stability under the harsh electrochemical regimes and conditions. Hence, it is clear that this report has not only offered the relatively facile and low-cost fabrication approach for the preparation of Ni-Co bimetal nanowires filled MWCNTs but has also paved the versatile avenue of novel nanocatalysts in highly sensitive, selective and durable non-enzymatic glucose sensor applications.

## Methods

### Materials

MWCNTs (length: 500–2000 nm; diameter: 20–30 nm), Tetraethyl orthosilicate (TEOS, reagent grade, 98%), cetyltrimethylammonium bromide (CTAB, AR, ≥99%), nickel(II) acetate tetrahydrate (Ni(CH_3_COO)_2_.4H_2_O, 98%), cobalt(II) acetate tetrahydrate (Co(CH_3_COO)_2_.4H_2_O, ACS reagent, ≥98%), sodium hydroxide (NaOH (pellet), AR, ≥98.5%), ethanol (HPLC, ≥99.8%), hydrochloric acid (AR, 35–37%), hydrazine monohydrate (N_2_H_4_.H_2_O, reagent grade, 98%), glucose (GC, 99.5%), ascorbic acid (AA, AR, ≥99.5%), uric acid (UA, HPLC, 99%), dopamine (DA, Ar, ≥99%), acetaminophen (AP, ≥99%), urea (U, AR, ≥99%), sodium chloride (NaCl, AR, ≥99.5%), citric acid (CA, AR, ≥99.5%) etc., were purchased from Sigma-Aldrich and used without any further purification.

### Preparation of Ni/Ni-Co nanowires filled MWCNTs

MSN/Ni, MSN/Co and MSN/Ni-Co nanostructures were prepared according to the procedures described elsewhere^49^. MSN/Ni/MSN/Co/MSN/Ni-Co catalyst (100 mg) was loaded into the alumina tubular furnace and purged with a nitrogen (N_2_) gas at a flow rate of 100 mL min^−1^ for 30 min. In a typical growth experiment, the acetylene gas was purged into the furnace with a flow rate of 60 mL min^−1^ at 750 °C at a heating rate of 5 °C/min for 30 min. Then the furnace was allowed to cool under the N_2_ atmosphere and the final product was collected. The percentage of carbon deposition due to the catalytic decomposition of acetylene was calculated by using the following equation^59^,


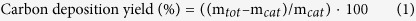


Where m_*tot*_ is the total mass of carbon product and catalyst and m_*cat*_ is the mass of catalyst. The obtained product was further sintered at 400 °C for 1 h in an atmospheric air to remove the carbonaceous impurities like amorphous carbon on the surface of MWCNTs, which was followed by the mild acid treatment for the removal of other impurities.

### Modification of electrodes

GCE with a surface area of 0.07 cm^2^ and a diameter of 3 mm was polished and dried according to the procedure described elsewhere^60^. The prepared nanostructure was homogenously dispersed in ethanol (1 mg ml^−1^) by using an ultrasonic treatment for 20 min, and the prepared slurry (6 μl) was coated on the GCE surface and dried at room temperature. The MWCNT/Ni, MWCNT/Co and MWCNT/Ni-Co nanostructures modified GCEs are designated as MWCNT/Ni/GCE, MWCNT/Co/GCE and MWCNT/Ni-Co/GCE, respectively. For the comparison, GCE was also modified with the commercially available MWCNTs (Pristine) and the corresponding electrode is represented as MWCNT/GCE.

### Characterizations

The prepared nanostructures were characterized by using a JEM-2100F transmission electron microscope (JEOL, Japan) equipped with EDAX analyzer, Riagaku X-ray diffractometer (XRD) and LabRamHR800 Raman spectroscopy. The four-probe method comprising Agilent multimeter, Cleveland, Ohio, U.S.A. was exploited to evaluate the electrical conductivities of prepared nanostructures.

### Electrochemical measurements

All the electrochemical experiments were performed by using a CHI-650D electrochemical workstation in a conventional three electrode cell containing GCE/modified GCEs as a working electrode, Pt wire as a counter electrode and Ag/AgCl as a reference electrode. The cyclic voltammograms of studied electrodes were recorded in 0.1 M NaOH at a potential scan rate of 20 mV s^−1^ in the absence and presence of 2 mM glucose. The amperometric responses of constructed electrodes were examined in 0.1 M NaOH with the continuous and gradual addition of different concentrations of glucose at an applied potential of 0.45 V *vs*. Ag/AgCl. The error bars were calculated from the standard deviations of five replicated measurements.

## Additional Information

**How to cite this article**: Ramachandran, K. *et al*. Ni-Co bimetal nanowires filled multiwalled carbon nanotubes for the highly sensitive and selective non-enzymatic glucose sensor applications. *Sci. Rep*. **6**, 36583; doi: 10.1038/srep36583 (2016).

**Publisher’s note:** Springer Nature remains neutral with regard to jurisdictional claims in published maps and institutional affiliations.

## Supplementary Material

Supplementary Information

## Figures and Tables

**Table 1:** The electrochemical quantification of glucose in human serum samples at MWCNT/Ni-Co/GCE.

Glucose Concentration		Glucose added (μM)	Glucose found (mM)		RSD^b^ (%)	Recovery (%)
Original serum sample (mM)	Diluted serum sample (mM)		Glucometer^a^	Proposed method		
9.60 (173 mg dL^−1^)	3.0	0	3.01	2.95	2.93	98.33
		100	3.10	3.07	2.09	99.03
		200	3.19	3.21	2.17	100.31
		300	3.29	3.32	2.14	100.61
		400	3.41	3.38	1.97	99.41

^a^Determined by *ACCU-CHEK* Active glucometer. ^b^Relative standard deviation of five measurements.
