# Supplementary material for: Ni-Co bimetal nanowires filled multiwalled carbon nanotubes for the highly sensitive and selective non-enzymatic glucose sensor applications
Source: Sci Rep. 2016 Nov 11;6:36583. doi: 10.1038/srep36583 (PMC5105137; doi:10.1038/srep36583)
Supplement: Supplementary Information [file srep36583-s1.pdf]

Ni-Co bimetal nanowires filled multiwalled carbon nanotubes for the highly sensitive and selective non-enzymatic glucose sensor applications

K. Ramachandran‡, T. Raj kumar‡, K. Justice Babu & G. Gnana kumar*

Department of Physical Chemistry, School of Chemistry, Madurai Kamaraj University, Madurai-625021, India. ‡These authors contributed equally to this work. Correspondence and requests for materials should be addressed to G.G (email: kumarg2006@gmail.com)

EDAX. The elemental composition of prepared nanostructures was analyzed by using EDAX analysis and the obtained EDAX patterns are shown in Fig. S1a,b. The EDAX pattern of MWCNT/Ni (Fig. S1a) confirmed that the prepared composite is composed of C (75.80 at %), O (19.64 at %) and Ni (4.56 at %). The presence of carbon peaks corresponds to the shells of nanotubes and the oxygen signals are attributed to the acid treatment. The presence of Ni(2.26 at %) and Co (2.22 at %) along with the C (75.62 at %) and O (19.90 at %) ensured the composition of MWCNT/Ni-Co and the average atomic ratio of Ni:Co is almost closer to the initial set ratio of Ni^{2+}:Co^{2+} = 1:1 (Fig. S1b).

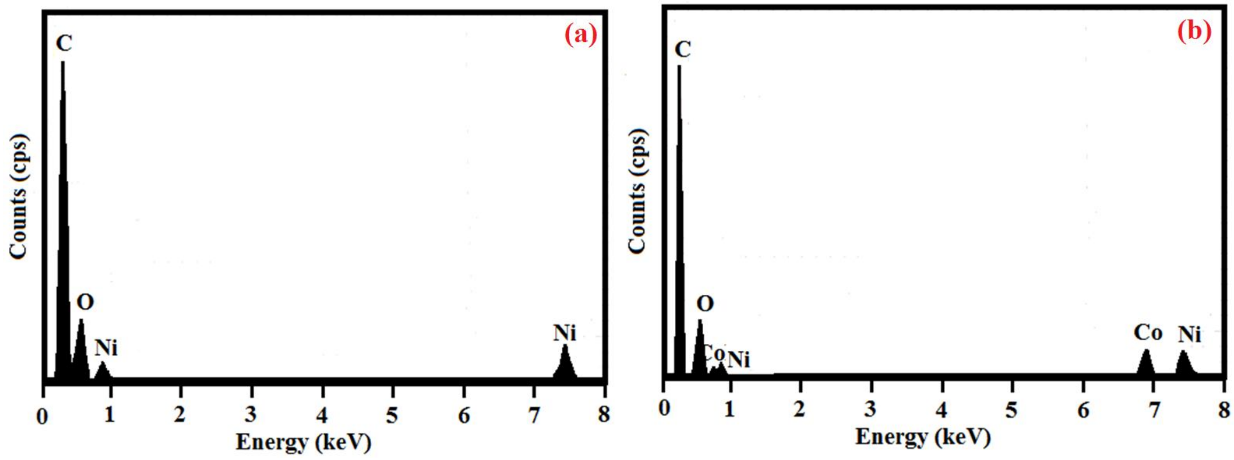

Figure S1. EDAX Patterns of **(a)** MWCNT/Ni and **(b)** MWCNT/Ni–Co nanostructures.

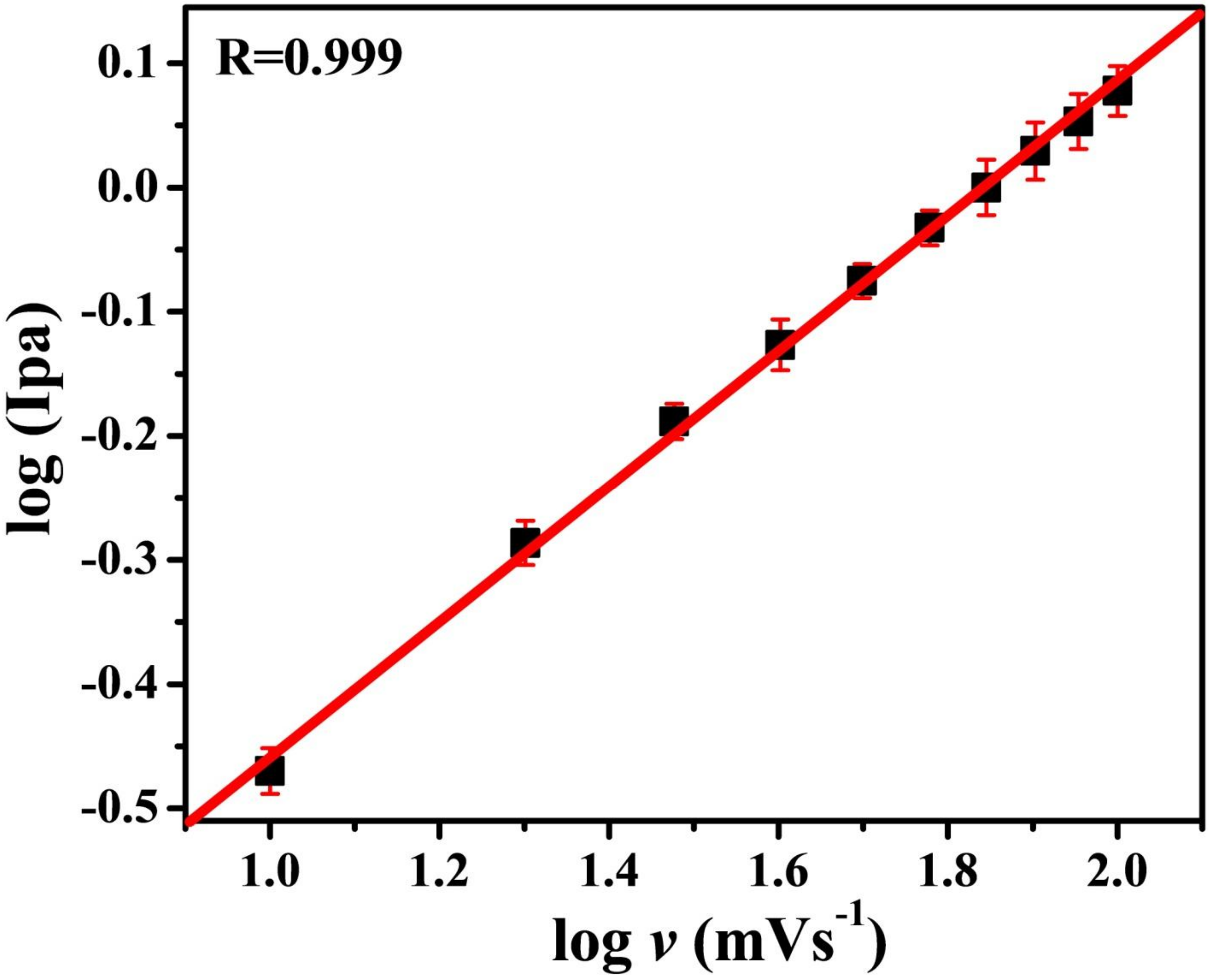

Figure S2. Plot of log Ipa *vs.* log *v* for 5 mM glucose in 0.1 M NaOH solution at MWCNT/Ni-Co/GCE with scan rate ranging from 10-100 mVs^{-1}

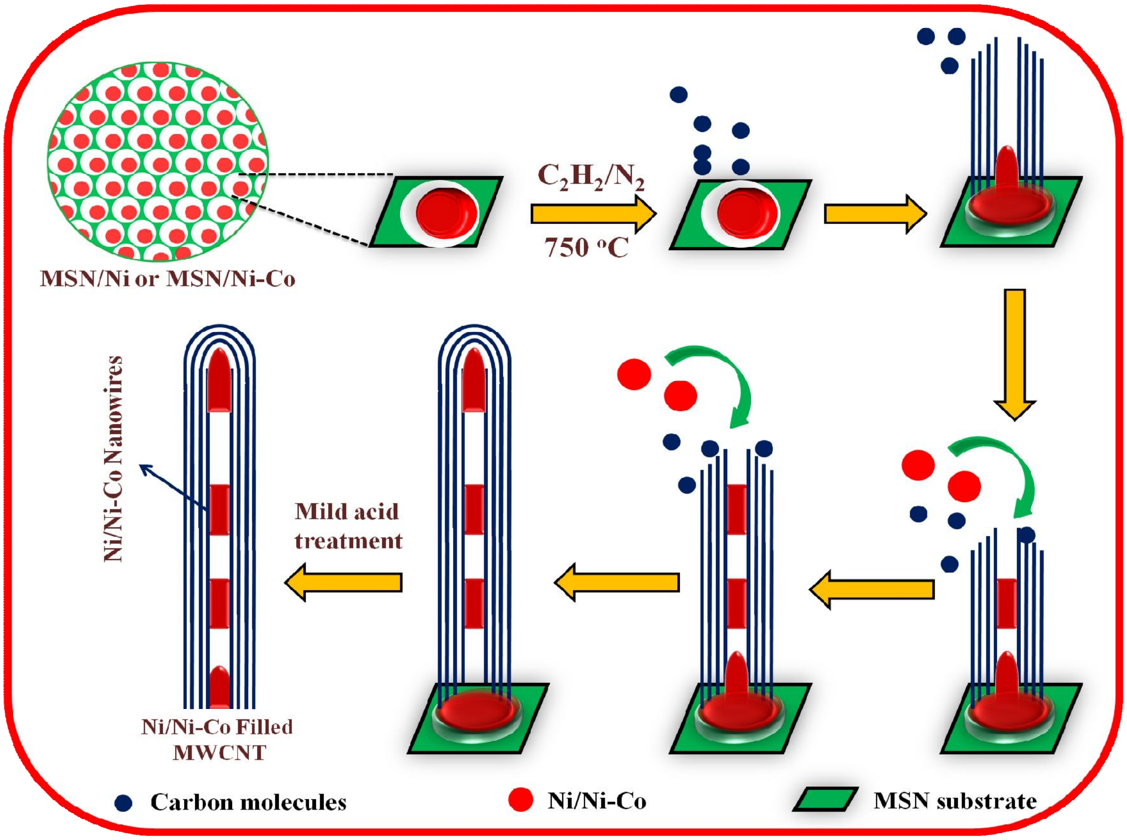

Figure S3. The schematic representation of mechanism involved in the growth of metal filled MWCNTs.

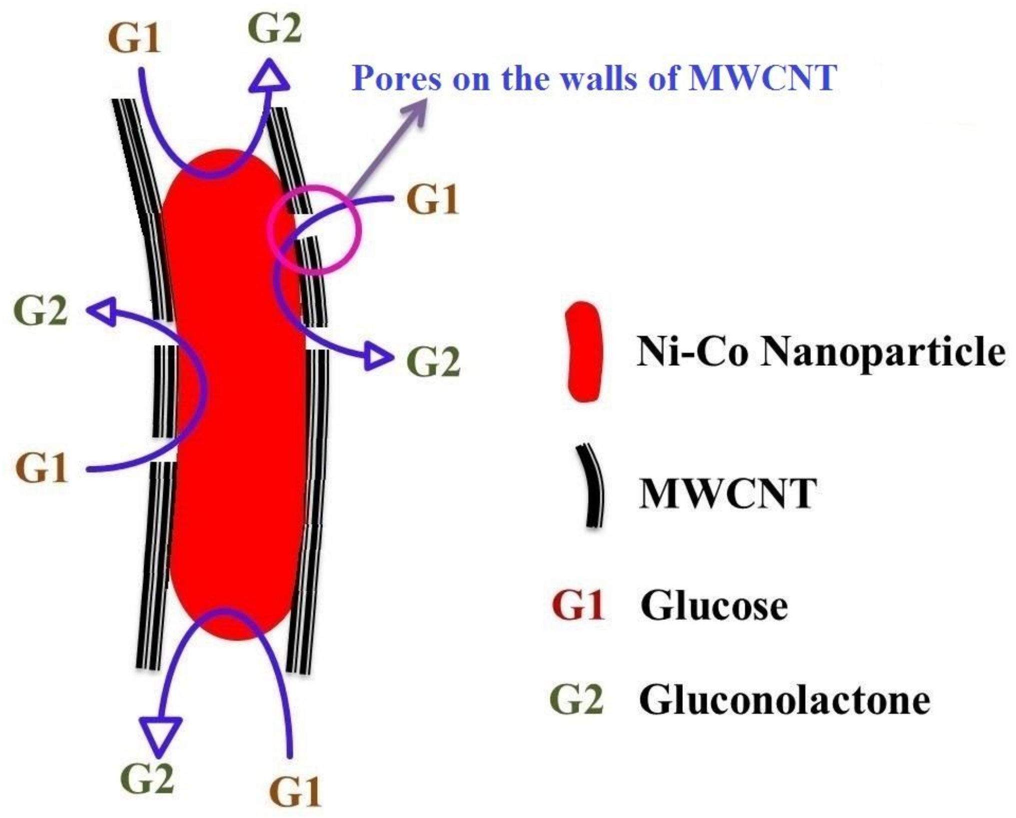

Figure S4. The schematic representation of mechanism involved in the adsorption and diffusion of glucose in the MWCNT/Ni-Co composite and the electroxidation of glucose.

Table S1. Comparison of the electroanalytical performances of non-enzymatic glucose sensors.

Electrode materials	Linear range (mM)	LOD[a] (μM)	Sensitivity ($\mu A mM^{-1} cm^{-2}$)	References
Zn/Co_3O_4	0.005 – 0.62	2.0	193.0	1
Co_3O_4-MWCNT[b]/GCE[c]	0.05-12.0	10.42	5089.1	2
CuCo-CFs[d]	0.02-11.0	1.0	507.0	3
Co_3O_4 NWs[e]	0.005 -0.57	5.0	300.8	4
Pd NCs[f]	1-10	-	34.0	5
Ni nanofoam	0.01-0.70	5.0	2370.0	6
CuO/MWCNTs[b]	0.004-14.5	4.0	1211.0	7
3D-Porous carbon-Ni NPs[g]	0.015-6.48	4.8	207.0	8
Ni-ITO[h]	0.02-3.0	3.74	610.0	9
CuNiO/graphene/GCE[c]	0.05–6.9	16.0	225.75	10
Fe_3O_4NPs[g]/MWCNT[b]/GCE[c]	0.5–7.0	15.0	238.7	11
Au-cluster film/FTO[i]	0.01–10.0	1.0	10.76	12
Nafion/Co_3O_4/GCE	0.001–0.3<br>4-12.5	0.1	471.5	13
MWCNTs-COOH-P2AT[j]-Au	0.1–30	3.7	1.40[k]	14
f-MWCNTs[l]/CuNSs[m]	0.01–6.91	1.53	1.39[n]	15
Co-MWCNT/S[o]	0.005–0.1<br>0.2–3.6	0.009<br>0.3	727<br>37	16
Ni-MWCNTs	0.0032 - 17.5	0.89	67.2	17
HPt[p]-CNT[q]s	0.0012–8.4	0.4	20.10[r]	18
MWCNT[b]/Ni/Co/GCE[c]	0.005-10.0	1.2	695.0	This work

[a] limit of detection; [b] multi-walled carbon nanotubes; [c] glassy carbon electrode; [d] carbon nanofibers; [e] nanowires; [f] nanocubes; [g] nanoparticles; [h] indium tin oxide layered dihydroxide; [i] fluorine doped tin oxide; [j] poly(2-aminothiophenol); [k] $\mu A\ mM^{-1}$; [l] functionalized multi-walled carbon nanotubes; [m] nanospheres; [n] $\mu M^{-1} cm^{-2}$; [o] mild steel substrate; [p] hollow nanoplatinum; [q] carbon nanotube; [r] $mA\ M^{-1}$

References :

1. Chowdhury, M., Cummings, F., Kebede, M. & Fester, V. Binderless solution processed Zn doped Co_3O_4 film on FTO for rapid and selective non-enzymatic glucose detection. *Electroanal.***28**, 1 – 10(2016).
2. Prasad R. & Bhat, B. R. Self-assembly synthesis of Co_3O_4/multiwalled carbon nanotube composites: an efficient enzyme-free glucose sensor. *New J. Chem.***39**, 9735—9742(2015).
3. Li, M. *et al*. Bimetallic MCo (M = Cu, Fe, Ni, and Mn) nanoparticles doped-carbon nanofibers synthetized by electrospinning for nonenzymatic glucose detection. *Sensor. Actuat B-Chem.***207**, 614–622(2015).
4. Kang, L., He, D., Bie, L. & Jiang, P. Nanoporous cobalt oxide nanowires for non-enzymatic electrochemical glucose detection. *Sensor. Actuat B-Chem.***220**, 888– 894 (2015).
5. Ye, J. S., Chen, C.W. & Lee, C.L. Pd nanocube as non-enzymatic glucose sensor. *Sensor. Actuat B-Chem.***208**, 569–574(2015).
6. Iwu, K. O., Lombardo, A., Sanz, R., Scirè, S. & Mirabella, S. Facile synthesis of Ni nanofoam for flexible and low-cost non-enzymatic glucose sensing. *Sensor. Actuat B-Chem.***224**, 764–771(2016).
7. Liu, X. W. *et al*. Ordered self-assembly of screen-printedflower-like CuO and CuO/MWCNTs modified graphite electrodes and applications in non-enzymatic glucose sensor. *J. Electroanal. Chem.***763**, 37–44(2016).
8. Wang, L. *et al*. A green and simple strategy to prepare graphene foam-like three-dimensional porous carbon/Ni nanoparticles for glucose sensing. *Sensor. Actuat B-Chem.* **239**, 172–179(2017).
9. Sivasakthi, P., RameshBapu, G. N. K. & Chandrasekaran, M., Pulse electrodeposited nickel-indium tin oxide nanocomposite as an electrocatalyst for non-enzymatic glucose sensing. *Mater. Sci. Eng. C.***58**, 782–789(2016).
10. Zhang, X. *et al*. CuNiO nanoparticles assembled on graphene as an effective platform for enzyme-free glucose sensing. *Anal. Chim. Acta* **858**, 49–54(2015).
11. Masoomi-Godarzi, S., Khodadadi, A. A., Vesali-Naseh, M. & Mortazavi, Y. Highly stable and selective non-enzymatic glucose biosensor using carbon nanotubes decorated by Fe_3O_4 nanoparticles. *J. Electrochem. Soc.***161(1)**, B19-B25(2014).
12. Han, L. *et al*. Porous gold cluster film prepared from Au@BSA microspheres for

electrochemical nonenzymatic glucose sensor. *Electrochim. Acta.***138**, 109–114(2014).

13. Han, L., Yang, D. P. & Liu, A. Leaf-templated synthesis of 3D hierarchical porous cobalt oxide nanostructure as direct electrochemical biosensing interface with enhanced electrocatalysis. *Biosens. Bioelectron.***63**, 145–152(2015).
14. Sedghi, R. & Pezeshkian, Z. Fabrication of non-enzymatic glucose sensor based on nanocomposite of MWCNTs-COOH-Poly(2-aminothiophenol)-Au NPs. *Sensor. Actuat B-Chem.***219**, 119–124(2015).
15. Chen, S. M., Devasenathipathy, R., Wang, S. F. & Kohilarani, K. Highly sensitive amperometric sensor for the determination of glucose at histidine stabilized copper nanospheres decorated multi-walled carbon nanotubes. *Int. J. Electrochem. Sci.***11**, 5416 – 5426(2016).
16. Premlatha, S., Sivasakthi, P. & Bapu, G.N.K.R. Electrodeposition of 3D hierarchical porous flower–like Cobalt-MWCNT nanocompositeselectrode for non-enzymatic glucose sensing.*RSC Adv.***5**, 74374-74380(2015).
17. Sun, A., Zheng, J. & Sheng, Q. A highly sensitive non-enzymatic glucose sensor based on nickel and multi-walled carbon nanotubes nanohybrid films fabricated by one-step co-electrodeposition in ionic liquids.*Electrochim. Acta***65,** 64-69(2012).
18. Wang, Y.*et al.* Direct electron transfer: Electrochemical glucose biosensor based on hollow Pt nanosphere functionalized multiwall carbon nanotubes.*J. Mol. Catal. B Enzym.***71**, 146-151(2011).
